# Integrating an exercise program into opioid agonist therapy: a pilot study on feasibility, fitness improvements, and participation challenges

**DOI:** 10.1186/s13722-025-00583-w

**Published:** 2025-07-08

**Authors:** Einar Furulund, Siv-Elin Leirvåg Carlsen, Silvia Eiken Alpers, Jørn Henrik Vold, Karl Trygve Druckrey-Fiskaaen, Tesfaye Madebo, Sindre M. Dyrstad, Torgeir Gilje Lid, Lars Thore Fadnes

**Affiliations:** 1 Centre for Alcohol and Drug Research, Stavanger University Hospital, Stavanger, Norway; 2 Department of Addiction Medicine, Bergen Addiction Research, Haukeland University Hospital, Bergen, Norway; 3 Department of Global Public Health and Primary Care, University of Bergen, Bergen, Norway; 4 Oral health Centre of Expertise Rogaland, Stavanger, Norway; 5 Department of Addiction Medicine, Haukeland University Hospital, Bergen, Norway; 6 Division of Psychiatry, Haukeland University Hospital, Bergen, Norway; 7 Department of Respiratory Medicine, Stavanger University Hospital, Stavanger, Norway; 8 Department of Clinical Science, University of Bergen, Bergen, Norway; 9 Department of Education and Sport Science, University of Stavanger, Stavanger, Norway; 10 Department of Public Health, University of Stavanger, Stavanger, Norway

**Keywords:** Substance-Related disorders, Mental health, Exercise therapy, Chronic disease

## Abstract

**Background:**

People receiving opioid agonist therapy (OAT) face high risk of comorbidities, including cardiovascular and mental health disorders. Integrating exercise programs with OAT may reduce health disparities and improve well-being. This study explored the feasibility and preliminary effects of an integrated exercise program.

**Method:**

This multicentre, mixed-methods pilot study was conducted in Western Norway, recruiting 22 participants receiving OAT from three outpatient clinics. The six-week, group-based exercise program focused on high-intensity endurance and strength training. Changes in aerobic fitness (4-minut step test), psychological distress (10-item Hopkins Symptom Checklist, SCL-10), fatigue (3-items Fatigue Severity Scale, FSS-3), lung function (spirometry), and respiratory symptoms (modified Medical Research Council Dyspnea Scale, mMRC) were assessed, while qualitative interviews provided insights into intervention feasibility.

**Results:**

Pre- and post-test assessments indicated improvements in aerobic fitness, as measured by the 4-minute step test, with a pre-test mean of 89.4 (SD: 24.7) and a post-test mean of 103.1 (SD: 31.3) step-cycles, despite a modest attendance rate of 28%. Psychological distress, evaluated using the SCL-10, increased in score from 1.85 (SD: 0.66) to 2.03 (SD: 0.59). Fatigue also increased slightly Fss-3: 4.44 (SD:2.38) to 5.07 (SD:2.01), while respiratory symptoms using mMRC, lung capacity with FVC, and expiratory volume FEV1 remained stable. Qualitative findings were categorized into three main themes: (1) The clinic as an arena for physical activity, (2) a modest move with a substantial benefit for participants, (3) challenges and adjustments to the intervention. Participants reported that exercising in connection with the OAT clinic promoted a sense of care and support from clinicians. Many experienced increased self-confidence and social engagement, though attendance was affected by health issues, fluctuating motivation, and logistical challenges.

**Conclusion:**

This pilot study suggests that integrating structured exercise into OAT can be feasible and improve aerobic fitness. However, increased psychological distress and fatigue might indicate the need for additional support. The low attendance rate highlights engagement challenges, emphasizing the need for tailored strategies to enhance participation. Future research should focus on optimizing intervention design to improve attendance and enhance physical and psychological outcomes for individuals in OAT.

**Supplementary Information:**

The online version contains supplementary material available at 10.1186/s13722-025-00583-w.

## Background

The life expectancy of individuals dependent on opioids is considerably reduced, with a loss of more than a decade compared to the general population [1]. This reduction is comparable to that observed in severe mental disorders, such as schizophrenia [2]. The decreased life expectancy among people with opioid dependence is attributed to various factors, such as opioid-related overdoses, suicides, injuries, and high morbidity from a range of chronic diseases including, mental health disorders [3]. Opioid agonist therapy (OAT) improves patients’ overall health and reduces mortality ( e.g., more than 50% reduction in the risk of all-cause mortality, drug-related deaths, and suicide) a substantial gap in health and life expectancy remains when compared to the general population, highlighting the need for further improvements in management [3, 4]. Retention rates for oral methadone and buprenorphine, both commonly used OAT treatments, are comparable ranging from 20 to 80%, with no significant differences between these treatments. However, low retention remains a challenge that requires further attention [5].

Regular exercise enhances cardiovascular fitness, reduces the risk of cardiovascular disease [6], and reduces depression and anxiety [7], which are common among individuals with opioid dependence and influence their physical, mental, or emotional well-being [8, 9]. To address these challenges, integrated treatment including multidisciplinary teams (of physicians, nurses, and social workers), could be beneficial. Reviews suggest that combining physical activity with traditional treatment for individuals in OAT could have substantial potential for improving overall health and well-being and reducing substance use, ultimately resulting in decreased morbidity and mortality [10–12]. Exercise could also be beneficial as an additional therapy in managing opioid use disorder by influencing the brain structure and neural pathways, particularly circuits involved in reward, control, and stress, which are key elements in addiction [13].

However, implementing physical activity interventions for individuals in OAT presents various challenges. Common barriers to exercise adherence include lack of motivation, health-related problems, or substance use [10, 14, 15]. Participation rates are often low and have been reported to range from 16 to 23% in several studies [16, 17]. Furthermore, physical activity interventions have varied in type and intensity, with most studies focusing on low- to moderate-intensity activities despite evidence suggesting that high-intensity activities could offer greater health benefits [11]. Integrating physical activity in OAT has shown improvements in physical fitness and reductions in substance use. However, mental health outcomes, such as reduction in anxiety and depression, are more inconsistent. Some studies report positive effects, while others show minimal impact [11, 18]. Given the variability in intervention types and the limited sample sizes of previous studies, more research is needed to establish standardized protocols, assess long-term impacts, and determine optimal intensity and dosing. Strengthening the evidence in this way could enhance adherence rates and improve both the physical and psychological health of individuals in OAT [11, 18].

The objective of this study was to pilot a six-week high-intensity exercise program for people receiving OAT and assess how exercise affected aerobic fitness, psychological distress fatigue and lung function. The study also investigated participants’ experiences, perceived barriers, facilitators, and the feasibility of the intervention.

## Method

### Design

This multicentre, mixed-methods pilot study evaluated an integrated exercise program in three outpatient clinics in Stavanger and Bergen, Western Norway. The exercise sessions were conducted in near the OAT clinics and facilitated by staff from the clinics. This study was designed from the start as a pilot study to assess the feasibility and preliminary outcomes.

### Participants and setting

In 2022, approximately 1300 individuals were administered OAT in Bergen and Stavanger, under the coordination of the involved clinics [19]. Two OAT clinics in Bergen and one in Stavanger participated in this pilot study, all shared similar characteristics. Each clinic employed a multidisciplinary team that consists of a consultant, a physician specializing in addiction medicine, nurses, social workers, and psychologists. Bergen has outpatient clinics spread across its suburbs, whereas Stavanger has a main central clinic and one for surrounding areas. Participants received OAT at their respective clinics (usually one to six times per week), or medication was delivered weekly by OAT personnel to their homes. Research nurses invited OAT patients to participate in the six-week exercise program.

The eligibility criteria for participation were as follows: (1) completed an annual health assessment, including the International Physical Activity Questionnaire (IPAQ) to assess physical activity levels, (2) delivering of OAT medication at least weekly, and (3) being 18 years of age or older. Exclusion criteria were: (1) planned hospitalizations or imprisonment during the intervention phase, (2) physical disabilities or behaviours that could hinder participation in group activities or the intervention itself (e.g., using crutches or experiencing a psychotic episode), and (3) current engagement in other organized exercise groups. As part of the recruitment strategy, we sought to reflect the demographic composition of Norway’s OAT population [20]. Ethics approval for the study was obtained from the Regional Committee for Health Research Ethics in South-Eastern Norway (registration no. 155386 REK southeast C, dated 23.09.2020/05.04.2022) All participants provided written informed consent before enrolment.

### Intervention

In this study, an integrated exercise program refers to the approach that combines exercise with the standard outpatient care provided by clinicians in extension of the clinical work. The exercise intervention consisted of three weekly supervised group-based outdoor sessions, with a maximum of 17 exercise sessions. No modifications were made to the intervention during the intervention period. Attendance was monitored throughout the intervention period to assess participation rates. The exercise sessions were conducted near the outpatient clinic, with participants either gathering inside or waiting outside before the start of each session. The sessions included both high-intensity endurance and strength training programs and lasted approximately 45 min. Each session started with a 15-minute endurance warm-up, followed by eight repetitions of intervals, 30 s of uphill high-intensity exercise (walking or running) and walking back to start as the resting period. The desired intensity of the session of > 5 on the Borg category ratio-10 [21] was randomly checked and adjusted (if necessary) by the research nurses. The session was completed with four strength training exercises at 30-second intervals, focusing on large muscle groups such as the pectoralis major, quadriceps femoris, gluteus maximus, and latissimus dorsi (detailed information on the session is presented in the Supplementary File). Participants with physical limitation, pain or discomfort were offered to optional strength training exercises during the session. These strength training exercises were performed in a fixed order. The structure remained consistent across the sessions to provide familiarity and facilitate participation.

As part of the intervention follow-up, research nurses sent short text message reminders one day before and on the morning of each session (see Supplementary File). The intervention followed the World Health Organization 2020 physical activity guidelines [22] and national chronic obstructive pulmonary disease guidelines [23] and was discussed with exercise therapist panels and user representatives at the University Hospitals in Bergen and Stavanger before the intervention. The exercise sessions were facilitated by a multidisciplinary team consisting of nurses, social workers and people with lived experience from OAT. Except for one facilitator, non-had prior experience in supervising exercise sessions or formal competence. To ensure a standardized approach, all facilitators received training on conducting the program and supervising participants. The training included guidance on monitoring exercise intensity using the Borg Scale, providing motivation and ensuring proper technique during both endurance and strength exercises. The exercise intervention sessions were conducted between May 19th, 2021, and June 25th, 2021. Although we did not systematically record weather conditions, the weather during the intervention period was generally favourable for outdoor activities, with only a few rainy days and mild temperatures. The intervention description followed the template for intervention description and replication (TIDieR); see the supplementary file.

To contextualize our intervention within existing research, we compared its characteristics with those of previous pilot studies on physical activity in OAT summarized in Supplementary Table S1. This comparison highlights differences in study design, supervision, intensity, and outcomes.

### Measures and data analysis

Pre-test outcomes were collected 1–2 weeks before the intervention began, except for SCL-10, where some data were extracted from annual health assessments conducted within the past six months prior to the intervention. Post-test outcomes were collected at the end of the intervention. The quantitative data were analysed using descriptive statistics such as percentages, mean and standard deviations using Stata statistical software 17 (StataCorp LLC). As this study was not designed for effect evaluation, no significance tests were performed. Validated measures were used for all outcomes. This included psychological distress, which was evaluated using the Norwegian-validated version of the Hopkins Symptom Checklist’s ten-item scale (SCL-10) [24]. The SCL-10 consists of ten items assessing symptoms of psychological distress. Each item is rated on 1–4 scale, where 1 indicates “not at all” and 4 indicates “very much”. The total score is calculated as the mean of all items, with higher scores reflecting greater psychological distress. Aerobic fitness was assessed through a 4-minute step test, which involved counting the number of completed steps [25]. In short: the participants stepped up and down from a 20-cm high step as fast as possible for 4 min and were allowed to pause and resume as often as needed. One full cycle of stepping up and down on the step box is counted as one step cycle. The Borg Scale-20 measured perceived exertion at the end of the step test [21]. Fatigue changes were measured using the three-item Fatigue Severity Scale (FSS-3) [26]. FSS-3 was used to measured general levels of fatigue, not specific related to the step-test or exercise sessions. The assessment of respiratory symptoms utilized the modified Medical Research Council Dyspnea Scale (mMRC), while lung function was evaluated through spirometry, specifically measuring forced vital capacity (FVC, indicating functional lung volumes for ventilation) and forced expiratory volume in 1 s (FEV1) [27].

For qualitative data collection, the interview guide was developed through a collaborative effort involving researchers, clinicians, and individuals with lived experience (see Supplementary Text). The main topics of the interview guide focused on participants experiences with the program, practical aspects, health and behavioural changes, and suggestions for future interventions. The interviews were conducted by EF and SELC at the participants affiliated clinic and the interviews lasted from 15 to 40 min. For qualitative data analysis, an inductive and thematic approach was used to analyse and describe key themes [28]. Of the 22 participants in the pilot study, 12 agreed to post-intervention interviews, all of whom had attended at least one session.

Following verbatim transcription, EF and SELC familiarized themselves with the transcripts. EF conducted the preliminary coding and thematic analysis, which SELC reviewed. SELC adjusted these codes and themes as necessary. In cases of discordance, a consensus between the two researchers was reached through discussion by agreeing on the proposed themes and codes or modifying them. NVivo 20 was employed for data analysis. Quotations presented in this article were translated to English after the analysis. Participants names are pseudonyms reflecting the person’s gender.

## Results

This study included 22 participants from three clinics, with 5,7, and 10 participants recruited from each site. The mean age of 51 years (SD: 10.2), of whom77% were male and 86% had completed either primary or high school education. At baseline, participants’ physical activity levels were generally low, and 50% had spirometry results indicating obstructive lung disease, defined as an FEV1/FVC ratio of less than 70% (see Table 1).

**Table 1:** Participants’ demographics and health characteristics

Characteristics	Frequencies (*N* = 22), (%), M (SD)
Age, Years	51 (SD 10.2)
Sex, female	5 (23%)
**Education**,** completed**	
No education	2 (9%)
Primary school (9 years)	10 (46%)
High school (12 years)	9 (41%)
College or university	1 (5%)
**Current living situation**	
Stable housing*	22 (100%)
Living alone	13 (59%)
**OAT medication**	
Methadone or morphine	12 (55%)
Buprenorphine	8 (36%)
Other	2 (9%)
**Health**	
Physical activity level**	
Low	12 (55%)
Moderate	10 (46%)
High	0 (0%)
Possible obstructive lung disease***	11 (50%)
**Mean age of onset for substance use debut ( in years)**	
Tobacco	13.9 (SD 4.0)
Alcohol	13.6 (SD 1.8)
Cannabis	16.6 (SD 5.2)
Benzodiazepines	21.3 (SD 8.3)
Stimulants	22.2 (SD 5.4)
Opioids	24.4 (SD 7.4)
**Substance use > 3 days a week**	
Tobacco	20 (91%)
Alcohol	3 (14%)
Cannabis	8 (36%)
Benzodiazepine	5 (23%)
Stimulants	2 (9%)
Opioids	0 (0%)

Note: Values are reported as the mean and standard deviation (SD) for continuous variables and n (%) for categorical variables. Percentages were calculated based on the total sample size (*n* = 22)

*Stable housing refers to stable, long-term living arrangements, including private residences, supported housing, or rentals, without immediate risk of eviction or homelessness

**Physical activity levels were assessed using the International Physical Activity Questionnaire short form, adhering to their guidelines for the evaluation of activity levels. Six participants reported some moderate intensity, but the determining factor for participants reaching a moderate activity level was walking > 600 Metabolic equivalents

*** Possible obstructive lung disease = if FEV_1_/FVC ratio < 70%

**Table 2:** Pre-and postintervention outcome measures (*n* = 15)

Tests	Pre test	Post test	Change
	Mean (SD)	Mean (SD)	Mean (SD)
Psychological distress (SCL-10)	1.85 (0.66)	2.03 (0.59)	0.18 (0.59)
Aerobic fitness (4 min step-test)	89.4 (24.7)	103.1 (31.3)	13.7 (14.4)
Test intensity (Borg-scale 20)	14.5 (1.92)	14.9 (2.69)	0.33 (2.23)
Fatigue (FSS-3)	4.44 (2.38)	5.07 (2.01)	0.62 (2.30)
Respiratory symptom score (mMRC)	1.23 (1.09)	1.30 (1.18)	0.07 (0.95)
Forced vital capacity (FVC)	4.32 (1.03)	4.44 (1.12)	0.12 (0.32)
Forced expiratory volume in 1 s (FEV1)	3.08 (1.07)	3.14 (1.07)	0.06 (0.17)

Note: Participants with valid scores on both the pre- and post-tests. Post-testing occurred on the final days of the intervention or soon afterwards. SD: Standard deviation. 4 min step-test data represent number of steps. Forced vital capacity and forced expiratory volume are reported in Liters

**Fig. 1:**
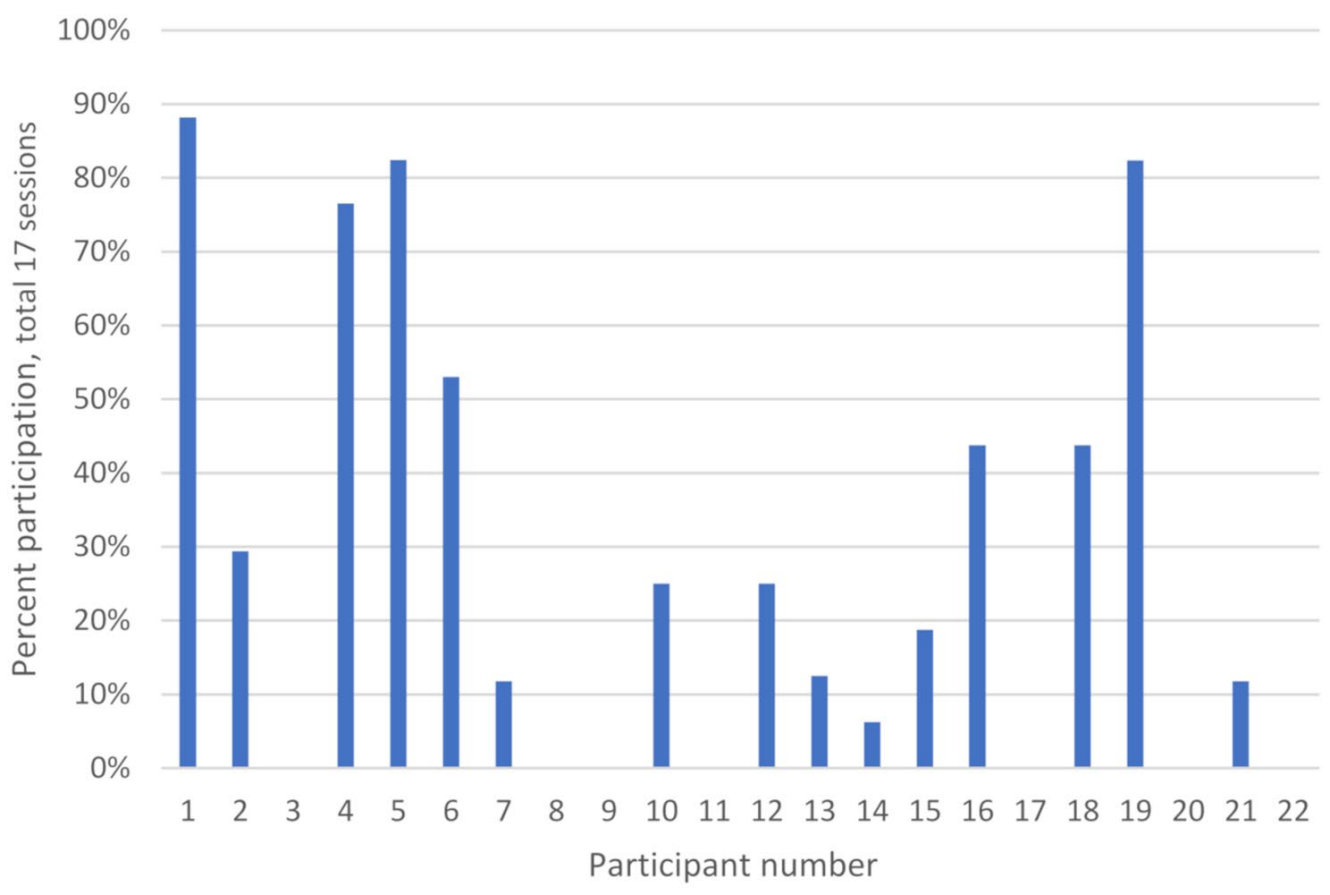
Attendance rates of participants in the exercise program in number of sessions

The mean attendance rate for the 22 participants included in the study was 28%, including 7 participants who intended to participate but did not attend any sessions (Fig. 1). Excluding non-participating individuals from the calculation, the attendance rate increases to 41%. 5 participants attended 50% or more of the offered sessions. Additional details on attendance categorized by age and sex, as well as comorbid conditions are available supplementary file. Table 3, reports explanations for participants lack of attendance. The mean Borg CR-10 score was 4.6 post-exercise suggesting moderate perceived intensity, with half of the participants reporting scores equal to or greater than this level (SD:1.50). There was a 15% increase in aerobic fitness measured with the step test, but a slight increase in psychological distress and fatigue during the intervention (Table 2). Measures for lung capacity and respiratory symptoms remained unchanged.

The follow-up qualitative interviews explored participants’ lived experiences with the intervention, delving into their perceptions, challenges, and reflections. This part aimed to capture the nuanced, individual perspectives that may have shaped their engagement with the intervention.

### The OAT clinic as an arena for physical activity ─ participants’ experiences

Several participants talked about an enhanced sense of care and attention from clinicians after the intervention, extending beyond mere medication delivery, which they attributed to the intervention. Engaging with clinical staff in a more informal and natural setting was particularly valued for improving their personal connections and openness, creating a more relaxed environment that facilitated discussions and conversations about other health concerns. A few participants found it convenient and motivating that the clinic offered the exercise sessions, as it allowed them to combine receiving medication with exercise Oscar reflected on this approach:

*“Hmm*,* yes*,* it shows they want us to get better. It is not just about giving us methadone […] because it is not just that simple. Unfortunately.”– Oscar*.

In the beginning, several participants felt uncomfortable and insecure about exercising and interacting with other participants. However, the supportive and welcoming environment established by the supervisors, including clinicians and peers with lived experience from OAT, helped ease these fears, some said. Several participants valued the group exercise sessions for building a sense of community through shared experiences, enjoyment, and collective goal achievement. Oscar, humorously recounted a moment of friendly competition during the session, saying:

*“I was walking fast [in the intervals]*,* and then another participant started to run. Then*,* he came beside me*,* so I had to start running too”.* - Oscar.

### A modest move with a substantial benefit for participants

Many participants reported a boost in their self-confidence during the intervention with some saying they could now hold their heads high while walking in the streets and were unafraid to speak with others. Others mentioned that they had become more socially engaged, had regular appointments again, and escaped from their anxiety and home confinement. Some participants also highlighted improved aerobic fitness, from walking during the intervals to running as the intervention progressed. In addition, some participants experienced that the intervention positively influenced other lifestyle factors. For example, some reported leaving their tobacco at home when participating in the exercise or postponing smoking until they returned home. However, a few participants worried about sustaining their newfound confidence and exercise habits after the program ended. In contrast, others felt motivated to continue exercising and sought to join physical activity groups. One participant emphasized the interventions’ role in promoting better health and reduced reliance on mobility aids. Bjørnar shared his journey of participating in the intervention:

*“At first*,* I thought it was too much for those of us who had been sitting at home for several years*,* just on the couch*,* right. And suddenly we were supposed to exercise three times a week*,* that was tough. Really tough. However*,* I made it through the first day and it was fun too. Then it [aerobic fitness] started to get a little better and a little better*,* and I walked faster and even started to run a bit. So*,* things have improved. And I’m happy about that.”.* - Bjørnar.

### Challenges and adjustments to the intervention

Several participants highlighted the critical role of peer and supervisors support in fostering motivation and engagement. The importance of receiving reminders through calls or text messages was also highlighted by some participants, who said they often forgot the appointment and found it nice that the research nurses called or sent them text messages. Moreover, concerns about a weakened sense of community, particularly during sessions with fewer attendees, affected the overall experience and motivation to participate. Even though there were different experiences, some found traveling and the timing of the session to be challenging. These posed as barriers to regular participation and complicated the integration of the program into their lives. Alan shared his experience on this:

*“I think the training could have started a bit earlier. Because it’s at 12:30*,* right*,* and then I might have been sitting around for two and a half hours*,* watching the clock*,* and waiting to leave home. The training could have started at 11 o’clock or something like that instead.” - Alan*.

Most participants were satisfied with the exercise program, although maintaining regular participation was challenging for many reasons, such as personal commitments, health issues, and logistical challenges. Some participants found the thrice-weekly schedule demanding, while others viewed it positively. Some also suggested to reduce the amount of running/walking and replace it with more time for strength training. A few participants also recommended moving some parts of the training indoors, especially strength exercises in cold or rainy weather, not just for comfort but also to avoid illness during COVID-19. Several participants said they had physical limitations, such as having a bad knee, hip, or back pain. They appreciated having alternative strength exercises and the ability to adjust according to their capabilities. Additionally, a few found it meaningful that the intervention also included pre- and post-tests, allowing them to see if there was progression. The intervention setting also played a role in participants’ experiences; some mentioned that they would have preferred conducting sessions in more discreet and less crowded environments. Both Tony and Thomas shared some thoughts on the program:

*“I liked that we always met at the same location and did similar exercises. That was nice*,* and I liked it because it was easy to get to and we meet here every week. In connection with these tests and such*,* it became a bit more meaningful in a way. Then we could see the progress.”– Tony*.

*“Some exercises are hard for me because of my foot and hip surgery. I think we can change some exercises and try new ones. Find something new to do. But right now*,* I’m at my limit*,* but I am okay with what we’re doing”.– Thomas*.

The explanations (Table 3) behind the varied attendance rates shed light on the complexities of participants’ lives and challenges with implementation. Two clinics reported having a functional group most of the intervention time. Many participants attended inconsistently, occasionally missing some sessions before returning to the group later. One clinic faced more challenges with attendance and recruitment, impacting overall participation. In addition to these implementation aspects, it was essential to contextualize the barriers posed by COVID-19, which may have created additional obstacles to consistent attendance during the intervention period.

**Table 3:** Participants’ explanations for their lack of attendance

Family and social commitments	Health issues	Medical appointments and related issues	Other reasons
-Trips with the family -Family matters -Dinner invitation -Meeting friends -Funeral -Boat trip with a friend -Illness in the immediate family -Helping relatives	-Not feeling well -Stomach trouble -Knee pain -Pain/swelling in the legs -Back pain -Stomach flu -Fatigue -Side effects after COVID-19 vaccine -Sleep problems	-Dentist appointments -GP appointments -Vaccination (COVID-19) -Medication (late delivery) -Medication (tapering, and irritation with the health system) -Medication (confusion with delivery) -Medication (not receiving benzodiazepines, was furious) -Social support appointment	-Did not receive SMS reminder -Imprisoned -Out of town -Overslept -Work Changes in housing situation

## Discussion

In our mixed-method pilot study, we explored the integration of a structured exercise program within an OAT setting to enhance the physical and mental health of individuals receiving OAT. Although attendance was modest, we observed improvement in aerobic fitness, consistent with findings from previous studies that report physical benefits of exercise interventions in similar settings [11]. The qualitative results showed that participants reported in the interviews that they felt more confident and had a more social life.

However, we observed a marginal increase in psychological distress and fatigue, which may be influenced by several methodological factors and initially low physical activity levels, as indicated by baseline scores. As this was a pilot study not powered for effect evaluation, statistical testing of effects was not an aim [29]. Consequently, the risk of type I and type II errors is less relevant, as the analyses were exploratory and primarily intended to inform feasibility and potential trends. Additionally, the short intervention may not have allowed sufficient time for potential psychological benefits to emerge. It is also possible that the SCL-10 questionnaire was not sensitive enough to capture changes within this study. The observed increases in fatigue and psychological distress may reflect several interacting factors, including social and relationship difficulties, chronic health conditions, and the demands of initiating exercise [3]. In non-randomized pilot studies, such outcomes may also be more sensitive to individual life events and variability. Furthermore, summarizing individual responses into a mean score may have masked potential differences across subdomains such as anxiety or depression. These findings contrast with meta-analyses that have generally shown physical activity to reduce symptoms of anxiety and depression [30, 31]. Nonetheless, the qualitative findings of improved self-confidence and social reintegration suggest that this may be part of an initial adjustment phase and should be interpreted with caution. Additionally, the slight increase in fatigue could be interpreted as a natural response to new physical exertion, a common occurrence during the early stages of lifestyle changes, which could decline over time as participants acclimate to their new lifestyle [32].

The BAReAktiv pilot study distinguishes itself from previous research through its integration of a structured, high-intensity exercise intervention within outpatient OAT settings, facilitated by both clinical staff and peer support personnel [11, 18]. With 22 participants over six weeks, the study is comparable in scale to other pilot interventions in this field, which typically range from 19 to 40 participants and last 8 to 16 weeks (see Supplementary). Although the shorter duration may limit impact on long-term psychological distress, it aligns with the study’s feasibility and implementation goals. Unlike most previous studies, which focused on light to moderate intensity activity and external supervision, our intervention used structured high-intensity exercise delivered by clinical staff within routine outpatient care [11]. This approach provides novel insights into the integration and practicality of such programs in clinical OAT, enhancing ecological validity and supporting potential for scale-up.

In line with previous research, this study identified several barriers to participation, including health problems, motivational challenges, and logistical difficulties [11, 33]. These barriers were addressed though an integrated clinical approach, which helped embed exercise into routine care, and by creating a safe and welcoming environment that encouraged participation. Additionally, continuous reminders of the sessions played a role in facilitating for attendance [34, 35]. Furthermore, our findings reflect the complexities of engaging in health-promoting behaviour, similar to previous studies that have highlighted inconsistent participation rates and the need for tailored strategies to improve adherence [12, 34, 36]. This variability in engagement emphasizes the need for future research to explore strategies that strengthen the effectiveness of exercise interventions, particularly in enhancing engagement and attendance. A promising approach might involve initial individual sessions between participants and supervisors to foster a sense of capability and commitment toward group activities [15]. Additionally, offering a variety of exercises and activities could help accommodate different abilities, preferences and motivation levels. Findings from the FitForChange trial suggest that integrating diverse physical activity options may improve adherence in individuals with substance use disorders [37, 38]. While our study focused on structured high-intensity endurance and strength training, integrating a more flexible exercise format could have been beneficial. However, offering a wider range of exercise may also place greater demands on the competence of the supervisors related to physical activity and exercise.

A key strength of this study was its integrated treatment approach, demonstrating the feasibility of incorporating an exercise program within an OAT setting. This integration shows potential for enhancing empirical evidence practice and treatment outcomes by including physical activity as a regular component in OAT. Integrating exercise into treatment plans for individuals with opioid dependence represents a more holistic approach that may improve outcomes and introduce new rehabilitation strategies, contributing to the shift of therapeutic focus from acute to chronic care [11, 12, 18, 39, 40]. However, the implementation of such interventions is challenging, even for motivated patients. The use of reminders, such as texts or calls, has emerged as a dual-purpose tool; in our study these reminders may both have improved attendance rates and contributed to a stronger sense of community within the group.

A limitation of this study is the absence of a control group, which in addition to the study size makes it difficult to evaluate the effect from this study [41]. Despite lacking a control group, the qualitative findings provide valuable insights into the participants’ lived experiences of the exercise intervention [42]. This complements the quantitative data by highlighting perceived improvements in well-being, barriers to participation, and potential ways to enhance the intervention. Future studies should include a control group to assess the specific effects of exercise on addiction outcomes [11]. One limitation of this study is the absence of data on addiction severity, which could influence the findings. Participants in this study presented with a high burden of health challenges, including comorbid substance use, mental health disorders, respiratory and cardiovascular conditions, and other somatic illnesses (see supplementary). This profile is consistent with international findings in OAT populations and highlights the complexity of addressing health behaviour change in this group [3]. When viewed alongside baseline indicators such as fatigue, reduced spirometry, and psychological distress, these comorbidities highlight the need for tailored interventions and a strong health-promoting focus.

The relatively low attendance rate of 28% may have contributed to variability in outcomes, as low or irregular attendance likely reduce the potential benefits of the intervention. This highlights the challenge of engaging this population in structured exercise programs and maintaining sustained participation, which can impact group dynamics and limit overall effectiveness of the interventions. The potential for monotony and its impact on attendance is also acknowledged as a possible limitation, as some participants may have benefited from greater variation in exercises [43]. The 4-minute step test used to measure aerobic fitness has clear limitations. It is a submaximal and indirect performance test and does not give a very reliable measure of participants’ aerobic fitness [44]. Factors such as motivation, technique, and the test’s learning effect could explain the test-retest difference. Nonetheless, the test is easy to perform, which minimizes the test’s learning effect, and no difference in perceived intensity was found between tests [25].

While the small sample size limits conclusions, some trends in attendance by age and gender were observed. Female participants were fewer in number and tended to have lower attendance overall. There was also considerable variation in attendance among male participants see figure in supplementary. Among the older participants (aged 60 and above), attendance tended to be lower, though this was observed across both sexes. It is possible that factors such as sex and age-related health or logistical challenges may have influenced participation, but further research with larger samples is needed to explore this. This study did not further evaluate the characteristics of those who did not participate in the exercise program. However as presented in supplementary demographic variables indicate that participants and non-participants were largely comparable. Exploring the characterises of the participants dropout in more detail could be an interesting for further research as seen in similar research involving individuals within depressive disorders and schizophrenia [45, 46]. Additionally, co-morbid chronic illnesses, common in these population may also influence attendance and need further examination.

The timing of Borg CR-10 score measurements, taken at the end of sessions after strength training rather than immediately after intervals, might not have accurately captured exertion levels, potentially affecting the exercise intensity data [47]. Research on session-rating of perceived exertion method suggest that the timing of collection can influence reported exertion, as delayed assessments my be affected by post-exercise recovery and memory bias [47]. Immediate post exercise ratings, particularly after the most intense segments of the session, might provide a more accurate reflection perceived exertion.

Also, restrictions limited by the COVID-19 pandemic, particularly limitations on group sizes (*n* < 10 in each group) and previous isolation, may have had impact on the study’s implementation and findings. Consequently, these findings should be interpreted with caution, considering the methodological limitation, including sample size, intervention duration and attendance variability. This pilot study was not designed or powered to assess intervention effects. Accordingly, we did not include significance tests such as *p*-values in our analysis. Nevertheless, this study provides important information relating to intervention experiences and feasibility of the intervention [48, 49].

## Conclusion

This pilot study reports that integrating structured exercise into OAT might be feasible for the participants and lead to measurable improvements in aerobic fitness. However, a slight increase in psychological distress and fatigue may indicate a need for additional support or slight adjustments to the intervention structure. The relatively low participation rate of below 30% signifies major challenges in engagement, highlighting the need for better strategies to improve adherence. While participants reported increased self-confidence and social interaction, future research should explore how to adjust program design to increase adherence and their potential in improving both physical and psychological outcomes. Addressing barriers to participation will be crucial in improving exercise interventions for individuals receiving OAT.

## Electronic supplementary material

Below is the link to the electronic supplementary material.

Supplementary Material 1

Supplementary Material 2

Supplementary Material 3

Supplementary Material 4

Supplementary Material 5

Supplementary Material 6

## Data Availability

Because of data protection regulations, the raw interview data for this study are not publicly available.

## Abbreviations

CR-10: Category Ratio-10 Scale (Borg Scale)
FEV1: Forced Expiratory Volume in 1 s
FSS: 3-Fatigue Symptom Scale
FVC: Forced Vital Capacity
IPAQ: International Physical Activity Questionnaire
mMRC: Modified Medical Research Council Dyspnea Scale
OAT: Opioid Agonist Therapy
SCL-10: Symptom Checklist’s Ten-Item Scale
SD: Standard Deviation

## References

[1] Lewer D, Jones NR, Hickman M, Nielsen S, Degenhardt L. Life expectancy of people who are dependent on opioids: A cohort study in new South wales, Australia. J Psychiatr Res. 2020;130:435–40. 32905957 10.1016/j.jpsychires.2020.08.013

[2] Hjorthøj C, Stürup AE, McGrath JJ, Nordentoft M. Years of potential life lost and life expectancy in schizophrenia: a systematic review and meta-analysis. Lancet Psychiatry. 2017;4(4):295–301. 28237639 10.1016/S2215-0366(17)30078-0

[3] Santo T Jr., Clark B, Hickman M, Grebely J, Campbell G, Sordo L, et al. Association of opioid agonist treatment with All-Cause mortality and specific causes of death among people with opioid dependence: A systematic review and Meta-analysis. JAMA Psychiatry. 2021;78(9):979–93. 34076676 10.1001/jamapsychiatry.2021.0976 PMC8173472

[4] Lindsay AP, Jeong Eun M, Micah P, Haoxuan Z, Fahmida H, Amanda S, et al. Opioid agonist treatment and risk of mortality during opioid overdose public health emergency: population based retrospective cohort study. BMJ. 2020;368:m772. 32234712 10.1136/bmj.m772 PMC7190018

[5] Klimas J, Hamilton M-A, Gorfinkel L, Adam A, Cullen W, Wood E. Retention in opioid agonist treatment: a rapid review and meta-analysis comparing observational studies and randomized controlled trials. Syst Reviews. 2021;10(1):216. 10.1186/s13643-021-01764-9 PMC8348786 34362464

[6] Kemi OJ, Wisløff U. High-Intensity aerobic exercise training improves the heart in health and disease. J Cardiopulm Rehabil Prev. 2010;30(1). 10.1097/HCR.0b013e3181c56b89 20040880

[7] Ben S, Timothy O, Rachel C, Dorothea D, Rosa V, Amanda W, et al. Effectiveness of physical activity interventions for improving depression, anxiety and distress: an overview of systematic reviews. Br J Sports Med. 2023;57(18):1203. 36796860 10.1136/bjsports-2022-106195 PMC10579187

[8] Saha TD, Kerridge BT, Goldstein RB, Chou SP, Zhang H, Jung J, et al. Nonmedical prescription opioid use and DSM-5 nonmedical prescription opioid use disorder in the united States. J Clin Psychiatry. 2016;77(6):772–80. 27337416 10.4088/JCP.15m10386 PMC5555044

[9] Katz C, El-Gabalawy R, Keyes KM, Martins SS, Sareen J. Risk factors for incident nonmedical prescription opioid use and abuse and dependence: results from a longitudinal nationally representative sample. Drug Alcohol Depend. 2013;132(1–2):107–13. 23399466 10.1016/j.drugalcdep.2013.01.010 PMC5408745

[10] Abrantes AM, Van Noppen D, Bailey G, Uebelacker LA, Buman M, Stein MD. A feasibility study of a peer-facilitated physical activity intervention in methadone maintenance. Ment Health Phys Act. 2021;21:100419. 34552664 10.1016/j.mhpa.2021.100419 PMC8452230

[11] Alpers SE, Furulund E, Pallesen S, Mamen A, Dyrstad SM, Fadnes LT. The role of physical activity in opioid substitution therapy: A systematic review of interventional and observational studies. Subst Abuse: Res Treat. 2022;16:11782218221111840. 10.1177/11782218221111840 PMC9280793 35845970

[12] Weinstock J, Wadeson HK, VanHeest JL. Exercise as an adjunct treatment for opiate agonist treatment: review of the current research and implementation strategies. Subst Abus. 2012;33(4):350–60. 22989278 10.1080/08897077.2012.663327 PMC4631114

[13] Psarianos A, Chryssanthopoulos C, Theocharis A, Paparrigopoulos T, Philippou A. Effects of a Two-Month exercise training program on concurrent Non-Opiate substance use in Opioid-Dependent patients during substitution treatment. J Clin Med. 2024;13(4):941. 38398255 10.3390/jcm13040941 PMC10888880

[14] Caviness CM, Bird JL, Anderson BJ, Abrantes AM, Stein MD. Minimum recommended physical activity, and perceived barriers and benefits of exercise in methadone maintained persons. J Subst Abuse Treat. 2013;44(4):457–62. 23199641 10.1016/j.jsat.2012.10.002 PMC3577996

[15] Uebelacker LA, Van Noppen D, Tremont G, Bailey G, Abrantes A, Stein M. A pilot study assessing acceptability and feasibility of Hatha yoga for chronic pain in people receiving opioid agonist therapy for opioid use disorder. J Subst Abuse Treat. 2019;105:19–27. 31443887 10.1016/j.jsat.2019.07.015 PMC6709876

[16] Cutter CJ, Schottenfeld RS, Moore BA, Ball SA, Beitel M, Savant JD, et al. A pilot trial of a videogame-based exercise program for methadone maintained patients. J Subst Abuse Treat. 2014;47(4):299–305. 25012555 10.1016/j.jsat.2014.05.007 PMC4487635

[17] Colledge F, Vogel M, Dürsteler-Macfarland K, Strom J, Schoen S, Pühse U, Gerber M. A pilot randomized trial of exercise as adjunct therapy in a heroin-assisted treatment setting. J Subst Abuse Treat. 2017;76:49–57. 28143679 10.1016/j.jsat.2017.01.012

[18] Jake-Schoffman DE, Berry MS, Donahue ML, Christou DD, Dallery J, Rung JM. Aerobic exercise interventions for patients in opioid maintenance treatment: a systematic review. Subst Abuse: Res Treat. 2020;14:1178221820918885. 10.1177/1178221820918885 PMC8832319 35153484

[19] Nesse L, Lobmaier P, Skeie I, Lillevold PH, Clausen T, Statusrapport. 2022. Første år med nye LAR-retningslinjer. [Status report for 2022. The first year of the new MAT guidelines]. Senter for rus- og avhengighetsforskning, SERAF; 2023.

[20] Waal H, Bussesund K, Clausen T, Lillevold PH. Kjønn og alder i LAR. Oslo: SERAF-rapport. 2018;1:2018.

[21] Williams N. The Borg rating of perceived exertion (RPE) scale. Occup Med. 2017;67(5):404–5.

[22] Bull FC, Al-Ansari SS, Biddle S, Borodulin K, Buman MP, Cardon G, et al. World health organization 2020 guidelines on physical activity and sedentary behaviour. Br J Sports Med. 2020;54(24):1451–62. 33239350 10.1136/bjsports-2020-102955 PMC7719906

[23] Gulsvik A, Lund J, Austgard E, Høegh Henriksen S, Langhammer A, Refvem O. Kols. Nasjonal Faglig Retningslinje Og veileder for forebygging, diagnostisering Og Oppfølging. Norwegian Directorate of Health; 2012.

[24] Strand BH, Dalgard OS, Tambs K, Rognerud M. Measuring the mental health status of the Norwegian population: a comparison of the instruments SCL-25, SCL-10, SCL-5 and MHI-5 (SF-36). Nord J Psychiatry. 2003;57(2):113–8. 12745773 10.1080/08039480310000932

[25] Vieira EB, Degani-Costa LH, Amorim BC, Oliveira LB, Miranda-Silva T, Sperandio PC, et al. Modified BODE index to predict mortality in individuals with COPD: the role of 4-Min step test. Respir Care. 2020;65(7):977–83. 31992673 10.4187/respcare.06991

[26] Vold JH, Gjestad R, Aas CF, Meland E, Johansson KA, Fadnes LT, Group I-HS. Validation of a three-item fatigue severity scale for patients with substance use disorder: a cohort study from Norway for the period 2016–2020. Health Qual Life Outcomes. 2021;19:1–11. 33653349 10.1186/s12955-021-01708-w PMC7923309

[27] Celli BR, Cote CG, Marin JM, Casanova C, Montes de Oca M, Mendez RA, et al. The Body-Mass index, airflow obstruction, dyspnea, and exercise capacity index in chronic obstructive pulmonary disease. N Engl J Med. 2004;350(10):1005–12. 14999112 10.1056/NEJMoa021322

[28] Braun V, Clarke V. Using thematic analysis in psychology. Qualitative Res Psychol. 2006;3(2):77–101.

[29] Thabane L, Ma J, Chu R, Cheng J, Ismaila A, Rios LP, et al. A tutorial on pilot studies: the what, why and how. BMC Med Res Methodol. 2010;10:1–10. 20053272 10.1186/1471-2288-10-1 PMC2824145

[30] Rebar AL, Stanton R, Geard D, Short C, Duncan MJ, Vandelanotte C. A meta-meta-analysis of the effect of physical activity on depression and anxiety in non-clinical adult populations. Health Psychol Rev. 2015;9(3):366–78. 25739893 10.1080/17437199.2015.1022901

[31] Schuch FB, Vancampfort D, Richards J, Rosenbaum S, Ward PB, Stubbs B. Exercise as a treatment for depression: A meta-analysis adjusting for publication bias. J Psychiatr Res. 2016;77:42–51. 26978184 10.1016/j.jpsychires.2016.02.023

[32] Rhodes RE, Sui W. Physical activity maintenance: A critical narrative review and directions for future research. Front Psychol. 2021;12. 10.3389/fpsyg.2021.725671 PMC8450373 34552537

[33] Matthews E, Fabian H, Gooney M, Rogers D, Firth J. An integrative overview of physical activity for people with opioid use disorder. Ment Health Phys Act. 2024:100651.

[34] Simonton AJ, Young CC, Brown RA. Physical activity preferences and attitudes of individuals with substance use disorders: A review of the literature. Issues Ment Health Nurs. 2018;39(8):657–66. 29505733 10.1080/01612840.2018.1429510

[35] Matthews E, Van Hout MC, Scheiben F, Cowman M. A qualitative study of physical activity and dietary practices of people accessing opioid agonist treatment in Ireland. Heroin Addict Relat Clin Probl. 2021;23(x):xx–xx.

[36] Lum A, Skelton E, Wynne O, Bonevski B. A systematic review of psychosocial barriers and facilitators to smoking cessation in people living with schizophrenia. Front Psychiatry. 2018;9. 10.3389/fpsyt.2018.00565 PMC6232499 30459658

[37] Gunillasdotter V, Andréasson S, Jirwe M, Ekblom Ö, Hallgren M. Effects of exercise in non-treatment seeking adults with alcohol use disorder: A three-armed randomized controlled trial (FitForChange). Drug Alcohol Depend. 2022;232:109266. 35033949 10.1016/j.drugalcdep.2022.109266

[38] Gunillasdotter V, Andréasson S, Hallgren M, Jirwe M. Exercise as treatment for alcohol use disorder: A qualitative study. Drug Alcohol Rev. 2022;41(7):1642–52. 36073088 10.1111/dar.13527 PMC9826429

[39] Thompson TP, Horrell J, Taylor AH, Wanner A, Husk K, Wei Y, et al. Physical activity and the prevention, reduction, and treatment of alcohol and other drug use across the lifespan (The PHASE review): A systematic review. Ment Health Phys Act. 2020;19:100360. 33020704 10.1016/j.mhpa.2020.100360 PMC7527800

[40] Weinstock J, Farney MR, Elrod NM, Henderson CE, Weiss EP. Exercise as an adjunctive treatment for substance use disorders: rationale and intervention description. J Subst Abuse Treat. 2017;72:40–7. 27666958 10.1016/j.jsat.2016.09.002 PMC5289308

[41] Whitehead AL, Sully BG, Campbell MJ. Pilot and feasibility studies: is there a difference from each other and from a randomised controlled trial? Contemp Clin Trials. 2014;38(1):130–3. 24735841 10.1016/j.cct.2014.04.001

[42] Creswell JW, Klassen AC, Plano Clark VL, Smith KC. Best practices for mixed methods research in the health sciences. USA: National Institutes of Health; 2011.

[43] Ryan RM, Deci EL. Overview of self-determination theory: an organismic dialectical perspective. Handb self-determination Res. 2002;2(3–33):36.

[44] Bennett H, Parfitt G, Davison K, Eston R. Validity of submaximal step tests to estimate maximal oxygen uptake in healthy adults. Sports Med. 2016;46:737–50. 26670455 10.1007/s40279-015-0445-1

[45] Stubbs B, Vancampfort D, Rosenbaum S, Ward PB, Richards J, Soundy A, et al. Dropout from exercise randomized controlled trials among people with depression: a meta-analysis and meta regression. J Affect Disord. 2016;190:457–66. 26551405 10.1016/j.jad.2015.10.019

[46] Vancampfort D, Rosenbaum S, Schuch FB, Ward PB, Probst M, Stubbs B. Prevalence and predictors of treatment dropout from physical activity interventions in schizophrenia: a meta-analysis. Gen Hosp Psychiatry. 2016;39:15–23. 26719106 10.1016/j.genhosppsych.2015.11.008

[47] Haddad M, Stylianides G, Djaoui L, Dellal A, Chamari K. Session-RPE method for training load monitoring: validity, ecological usefulness, and influencing factors. Front NeuroSci. 2017;11. 10.3389/fnins.2017.00612 PMC5673663 29163016

[48] Teresi JA, Yu X, Stewart AL, Hays RD. Guidelines for designing and evaluating feasibility pilot studies. Med Care. 2022;60(1):95–103. 34812790 10.1097/MLR.0000000000001664 PMC8849521

[49] Leon AC, Davis LL, Kraemer HC. The role and interpretation of pilot studies in clinical research. J Psychiatr Res. 2011;45(5):626–9. 21035130 10.1016/j.jpsychires.2010.10.008 PMC3081994

