# Supplementary material for: Integrating an exercise program into opioid agonist therapy: a pilot study on feasibility, fitness improvements, and participation challenges
Source: Addict Sci Clin Pract. 2025 Jul 8;20:52. doi: 10.1186/s13722-025-00583-w (PMC12235965; doi:10.1186/s13722-025-00583-w)
Supplement: Supplementary file 1 — Supplementary Material 1 [file 13722_2025_583_MOESM1_ESM.pdf]

Examples of text-messages sent to participants

Typical group messages

- Welcome to the training today at 13:30. Remember, rain ware 🌧.

  Best regards

  Research nurse name

- Welcome to the activity group today at 13:00. The weather is glorious for training 🙂.

  Kind regards

  Research nurse name

Typical messages to participants who did not meet the last session

- Hi

  Are you ready to do some exercises today at 13:00? Would you like me to meet you outside ten minutes prior to the start?

  Best regards

  Research nurse name

- Hi
  Welcome to the activity group today at 1 p.m. We met at the bench we agreed on last time. If in doubt, just call 🙂. Hope you will come (participants name),

  with kind regards

  Research nurse name
