# Supplementary material for: Integrating an exercise program into opioid agonist therapy: a pilot study on feasibility, fitness improvements, and participation challenges
Source: Addict Sci Clin Pract. 2025 Jul 8;20:52. doi: 10.1186/s13722-025-00583-w (PMC12235965; doi:10.1186/s13722-025-00583-w)
Supplement: Supplementary file 2 — Supplementary Material 2 [file 13722_2025_583_MOESM2_ESM.pdf]

Interview guide

Q: What were your expectations for participating in the project?

Q: What motivated you to participate?

Q: What are your main takeaways from participating in BAReAktiv?

Q: Was there anything you found helpful/positive about participating?

Q: Was there anything you found negative/harmful/problematic?

Q: Did you participate in everything – why/why not?

*Follow up on any themes from initial responses.

Q: What do you think about the practical aspects of the training (such as timing, location, group size/composition, relationship with the instructor, etc.)?

Q: What do you think about the training sessions themselves? (Intensity, duration, motivational, enjoyable?)

Q: What is your current exercise/activity habits?

Q: How did you perceive the engagement of the project's instructor? How did it impact you and your participation?

Q: Has participating in the project changed the way you think about your own health?

Q: Has participating in the project led you to make changes in your daily life? If so, what changes?

Q: With the experience you have from the project you participated in, what are your thoughts on your exercise/smoking habits/diet going forward?

Q: How did you find the length of the project (appropriate/too short/long, other)?

Q: Is there anything you think should have been done differently/anything you missed in the program?

Q: Going forward, how will you manage to maintain the changes you have made?

Q: Are there any changes/possible other changes you think will be relevant for you going forward? (What do you need to maintain this?)

Q: For smokers: Have you made any changes in your smoking habits?

Q: Is there anything else you'd like to add or anything on your mind that I haven't asked about?
