# Supplementary material for: Integrating an exercise program into opioid agonist therapy: a pilot study on feasibility, fitness improvements, and participation challenges
Source: Addict Sci Clin Pract. 2025 Jul 8;20:52. doi: 10.1186/s13722-025-00583-w (PMC12235965; doi:10.1186/s13722-025-00583-w)
Supplement: Supplementary file 4 — Supplementary Material 4 [file 13722_2025_583_MOESM4_ESM.pdf]

This material is used with permission from www.exorlive.com

Participants who are unable to complete 3 sets are encouraged to do their best. It is okay not to manage 3 sets.

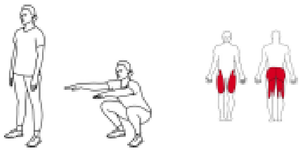

1. Squats

Stand with your feet shoulder-width apart. Bend down to about 90 degrees at the knees and press back up. Keep your back straight and your gaze forward. Alternatively, you can hold the deep squat position for a few seconds before pressing back up.

Sett: 3 , Varighet: 30 sek

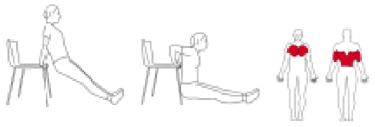

2. Dips på en benk / eller lignende

Dips on a bench / or similar:
Support yourself on the bench with a grip about shoulder-width apart. Keep your body almost straight so that only the heels are on the floor. Bend at the elbows and lower your body towards the floor. Press back up.

The closer your body is to your heels, the easier the load will be

Sett: 3 , Varighet: 30 sek

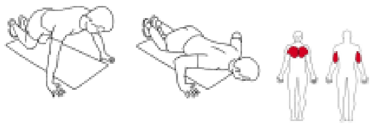

3. Push-up on knees / regular / against bench:

Position yourself on your knees with a fixed body. Place your hands wider than shoulder-width apart. Lower your body towards the floor and press back up without bending at the hips.

Sett: 3 , Varighet: 30 sek

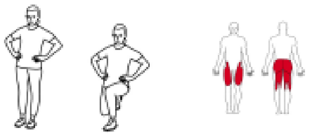

4. Lunges

Stand with your feet together and place your hands on your hips. Lift one leg and step forward. When your foot hits the floor, slow the movement until your body is in a deep position. Keep your upper body straight. Push off and move on to the next step. Knee control and a neutral back are important throughout the entire movement.

Sett: 3 , Varighet: 30 sek

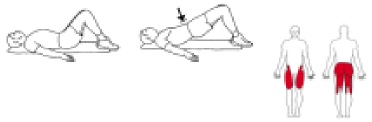

5. Hip lift (Optional exercise)

Place your feet flat on the floor and arms out to the sides. Tilt your pelvis backward. Engage the lower and deepest part of your abdominal muscles, pulling your navel toward your spine to create a "flat stomach." Lift your pelvis off the floor until you are resting on your shoulder blades. Lower and lift slowly.

Sett: 3 , Varighet: 30 sek

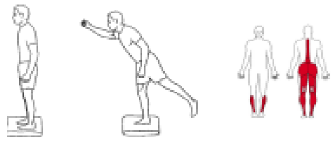

6. Standing diagonal lift on a mat (Optional exercise)

Stand on the mat, lift one leg, and bend slightly forward at the hip. Stretch the active leg backward and lift the opposite arm forward while maintaining balance. Repeat on the opposite side. Keep your back straight the entire time.

Sett: 3 , Varighet: 30 sek
