# Supplementary material for: Integrating an exercise program into opioid agonist therapy: a pilot study on feasibility, fitness improvements, and participation challenges
Source: Addict Sci Clin Pract. 2025 Jul 8;20:52. doi: 10.1186/s13722-025-00583-w (PMC12235965; doi:10.1186/s13722-025-00583-w)
Supplement: Supplementary file 5 — Supplementary Material 5 [file 13722_2025_583_MOESM5_ESM.pdf]

Supplementary table S1: Summary of pilot studies on exercise-based intervention in opioid agonist therapy populations.

Study	Design	Population & setting	Intervention	Supervision	Intensity	Main outcomes variables	Key findings
BAReAktiv, Present study (Norway)	Mixed-methods, multicentre pilot study	N = 22, Opioid agonist therapy outpatients.	6-week integrated exercise program. Offered 3x/week, endurance & strength training.	Supervised by research nurses and, clinic staff & peer support.	Vigorous intensity	Feasibility, attendance, psychological distress, fatigue, aerobic fitness, lung function	Positive feedback on intervention and implementation, moderate attendance. Positive trends in physical fitness.
Abrantes et al. 2021. (USA)	Single-arm feasibility study	N = 26, Outpatients in methadone maintenance treatment.	12-week program with 1x 20-30 min discussions, and 1x 30 min walking sessions.	Trained peers led the sessions.	Light intensity	Substance use, mental health, physical activity/ fitness, Satisfaction & perceived benefit, safety & adverse events.	Small to moderate reduction in illicit substance use. No significant mental health changes. 63% adherence of the intervention.
Colledge et al. 2017. (Switzerland)	Randomized controlled single-blinded pilot trial	N = 24, Outpatients in heroin-assisted treatment.	12-week intervention, 2x sessions weekly. Mixed physical activities (climbing, badminton, strength, boxing, dance, walking).	Trained research staff with background from sports education	Moderate to vigorous intensity	Substance use, physical and mental health, physical fitness and activity,	No significant substance use reduction and mental health effects but improved physical activity. 38% attended ≥80% of sessions, 54%

							attended 20–80%, and 8% attended <20%
Cutter et al. 2014. (USA)	Randomized controlled pilot trial	N = 29, Outpatients in methadone maintenance treatment.	8-week, 5 weekly sessions of 20-25 min/session. Video game (Wii Fit Plus) different activity games.	A member of the research team conducted an orientation.	Light intensity	Substance use, mental health, physical activity and fitness, acceptability	High acceptability of Wii Fit Plus, with 63% adherence and 7% dropout. Reduced drug use and stress in both groups, with no significant differences.
Pérez-Moreno et al. 2007. (Spain).	A randomized controlled single-blinded pilot study	N = 19, inmates in methadone maintenance treatment. In prison setting.	16-week of 3 weekly sessions, 90 min/session. Aerobic and strength training.	Supervised by the research personnel.	Moderate, gradually increased during the intervention, up to 80% of Hart rate max.	Substance use, physical and mental health, physical activity/fitness	Quality of life improved in exercise group, improved physical fitness, methadone dosage decreased in both groups.
Uebelacker et al. 2019. (USA).	Randomized controlled pilot trial	N = 40, Outpatients in opioid maintenance therapy.	12-week, 1 session each week of 1h Yoga.	Registered yoga teachers	Light intensity	Physical & mental health, Physical activity/ fitness, Safety & adverse events, intervention perception and satisfaction	50% adherence, 10% dropout. 61% of the yoga group practiced at home, but class attendance was lower than desired. Acceptable

							satisfaction levels. anxiety and pain reduction in the yoga group.

Note: Exercise intensity levels are classified based on reported study data, including qualitative descriptions (light/moderate/vigorous).

Table s2: Demographic characteristics of Participants and Non-Participants

Demographic information	Participants (N=15) M (SD) or (%)	Non-participants (N=7) M (SD) or (%)
Age	51 (11)	50 (10)
Sex, female	4/15 (26.7%)	1/7 (14.3%)
Permanent living conditions*	15 (100%)	7 (100%)
Mean age of first substance use (Years)		
Tobacco	13.2 (2.4)	15.6 (6.0)
Alcohol	13.8 (1.6)	13.1 (2.1)
Cannabis	15.4 (2.5)	19.0 (8.2)
Stimulants	21.3 (3.9)	24.4 (1.9)
Benzodiazepines	20.7 (6.4)	22.6 (12.7)
Opioids	25.3 (8.9)	22.7 (3.2)
Health		
Possible obstructive lung disease**	7 (46.7%)	4 (57.1%)

**Permanent living conditions = stable, long-term housing (not homeless or institution).*

*** Possible obstructive lung disease= cut-off < 70% for the FEV_1/FVC ratio.*
