# Supplementary material for: Integrating an exercise program into opioid agonist therapy: a pilot study on feasibility, fitness improvements, and participation challenges
Source: Addict Sci Clin Pract. 2025 Jul 8;20:52. doi: 10.1186/s13722-025-00583-w (PMC12235965; doi:10.1186/s13722-025-00583-w)
Supplement: Supplementary file 6 — Supplementary Material 6 [file 13722_2025_583_MOESM6_ESM.pdf]

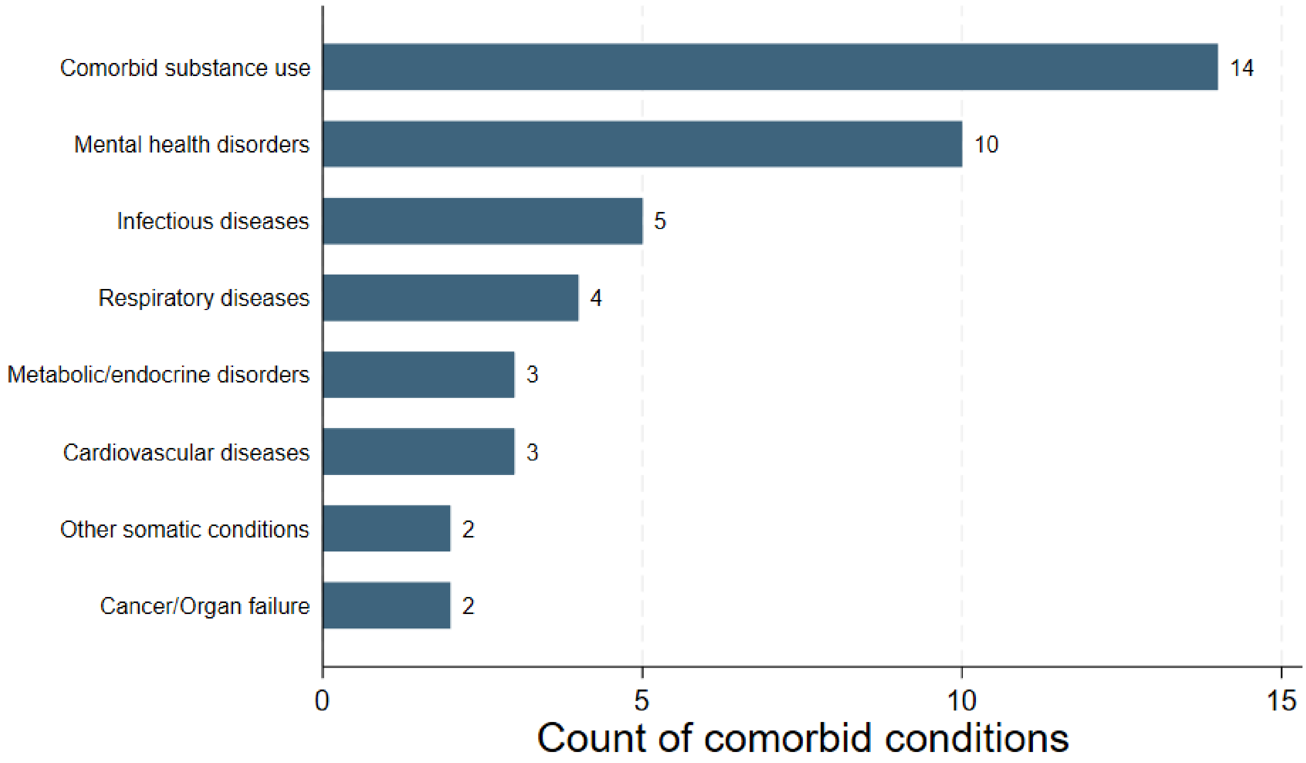

Figure: Prevalence of comorbid conditions

Note: Based on data from *n = 22* participants. *Comorbid substance use includes harmful use or dependence syndrome related to one or more of the following substances: alcohol, cannabis, benzodiazepines, stimulants, and nicotine. Mental health disorders include depressive disorders, psychotic disorders (e.g., schizophrenia and substance-induced psychosis), anxiety disorders, post-traumatic stress disorder, and eating disorders such as anorexia nervosa. Respiratory diseases include asthma, chronic obstructive pulmonary disease, emphysema, and respiratory failure. Infectious diseases include HIV infection, hepatitis C, sepsis, abscesses, and infective endocarditis. Cardiovascular diseases include angina pectoris, bradycardia, and hypotension. Metabolic and endocrine disorders include type 2 diabetes mellitus, hypothyroidism, and testicular hypofunction. Cancer and organ failure includes prostate cancer and liver failure. Other somatic conditions include anaemia and gastritis.*

Table: Mean (SD) attendance and age by sex

**Sex**	**Number participants**	**Attendance (Mean ± SD)**	**Age (Mean ± SD)**
**Female**	5	3.0 ± 2.7	49.7 ± 11.0
**Male**	17	5.1 ± 5.7	51.1 ± 10.3

Note: Mean attendance and age are presented with standard deviations (±SD) for male and female participants.

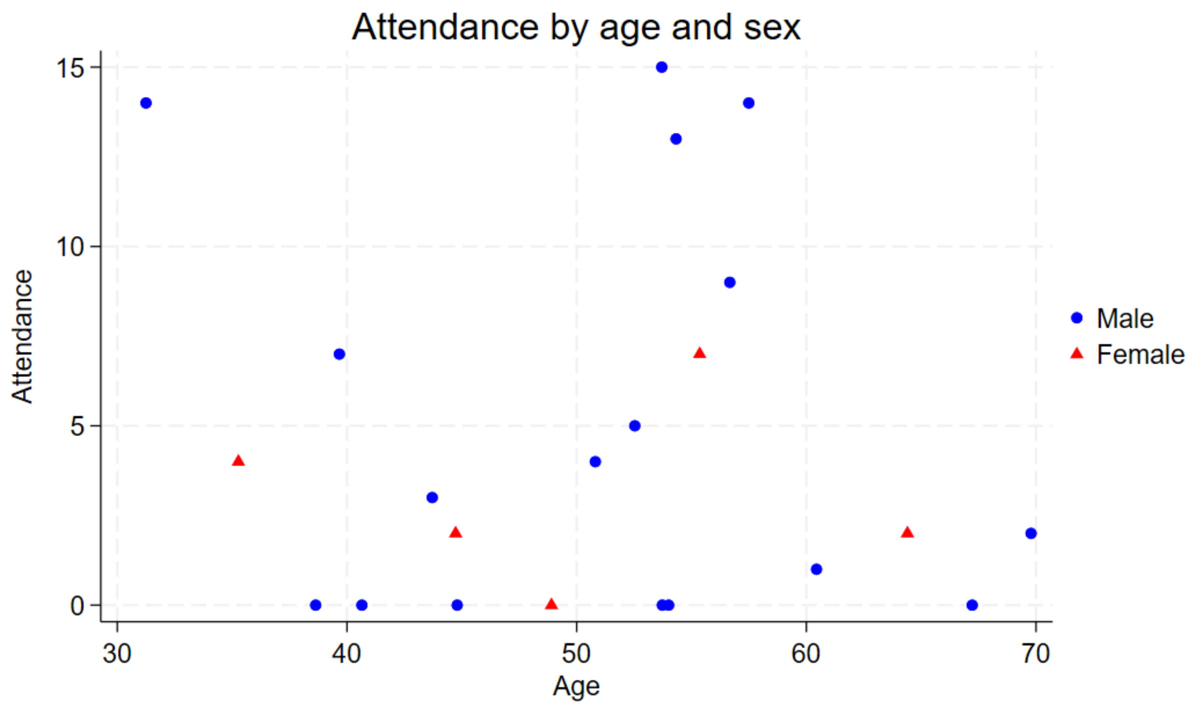

Figure: Attendance by age and sex

Note: Each point represents an individual participant's attendance in relation to their age and gender. Blue circles indicate male participants, and red triangles indicate female participants.
